# Clinicopathological correlates of vitamin D receptor expression in prostate cancer: results of genomic analysis

**DOI:** 10.1097/j.pbj.0000000000000280

**Published:** 2025-02-17

**Authors:** Sebastian A. Omenai, Henry O. Ebili, Uchenna S. Ezenkwa, Ayotunde O. Ale, Patrick A. Akintola, Adesoji E. Adetona, Chima U. Akunwata, Mbwas I. Mashor, Ifeanyichukwu D. Nwanji, Oluwadamilare Iyapo, Chinedu A. Ezekekwu, John C. Akulue, Ngozi Chidozie, Ifeanyi J. Nwadiokwu

**Affiliations:** a Department of Anatomical Pathology, Edo State University, Iyamho Uzairue, Nigeria; b Morbid Anatomy and Histopathology Department, Olabisi Onabanjo University, Sagamu, Ogun State, Nigeria; c Department of Anatomic Pathology, Federal University of Health Sciences, Azare, Bauchi State, Nigeria; d Medicine Department, Olabisi Onabanjo University, Sagamu, Ogun State, Nigeria; e Department of Haematology, University College Hospital, Mokola, Ibadan, Oyo State, Nigeria; f Department of Morbid Anatomy, Bingham University, Jos Campus, Jos, Plateau State, Nigeria; g Department of Pathology, University College Hospital Ibadan, Mokola, Ibadan, Oyo State, Nigeria; h Department of Morbid Anatomy, Eko University of Medicine and Health Sciences, Ijanikin, Lagos; i Bristol Haematology and Oncology Centre, University Hospitals Bristol and Weston NHS, Bristol, United Kingdom; j Department of Haematology, Alex Ekwueme Federal University Teaching Hospital, Abakaliki, Ebonyi State, Nigeria; k Acute Medical Unit, Basildon and Thurrock University Hospitals NHS Foundation Trust, Basildon, United Kingdom; l Department of Anatomic Pathology and Forensic medicine, Babcock University Teaching Hospital, Ilisan, Ogun State

**Keywords:** prostate cancer, vitamin D receptor expression, vitamin D receptor regulation, Gleason score, ISUP grade

## Abstract

**Objectives:**

Prostate cancer (PCa) is the most common malignancy in men. Geography and environmental factors have been associated with varying incidence and mortalities in different groups. Vitamin D has antiproliferative effect on PCa cells, and its effect is mediated through vitamin D receptor (VDR). This study reported the correlation of VDR expression with some clinicopathological and biological features among a cohort of patients with PCa.

**Methods:**

Genomic and clinicopathological data of 497 patients with PCa reposited in The Cancer Genome Atlas were retrieved using Linux command in running codes and scripts and extrapolated onto SPSS version 28 for statistical analysis. Descriptive and inferential statistics were conducted to determine the proportions and associations of VDR expression with genomic variables and clinicopathological indices. The mechanism of VDR dysregulation was also interrogated.

**Results:**

Our results showed that high VDR expression was positively correlated with a high Gleason score (*P* < 0.001), poorer prognostic International Society of Urological Pathology grade groups (*P* < 0.001), advanced tumor stage (*P* = 0.01), and poorer response to androgen deprivation therapy (ADT). Age, race, and overall and disease-free survival did not show any correlation with VDR expression (*P* > 0.05). Furthermore, the major mechanism of dysregulation of VDR in PCa was by aberrant methylation of the VDR promoter region (*P* < 0.001), and not by copy number alterations (*P* = 0.42).

**Conclusion:**

VDR expression is associated with adverse clinicopathological indices, including late-stage disease profile, high-grade indices, and poorer response to ADT. VDR is also mainly deregulated by aberrant epigenetic mechanism. The study is limited by absence of some clinical information such as sunlight exposure.

## Introduction

Prostate carcinoma is common in men as they grow in age.^1^ Globally, it is a common malignancy with significant mortalities.^2^ It ranks as the most common cancer in men in most Western countries and Sub-Saharan Africa, with an age-standardized incidence rate of 19.1 per 100,000 men population in Nigeria.^2,3^ The incidence of prostate cancer (PCa) is highest in African Americans worldwide, whereas in Asia, Japanese and Chinese men have the lowest rates.^4^

A study in the United States showed that mortality rates are higher in the northern part of the country while rates are higher among African Americans compared with Nigerians.^5,6^ Various explanations have been adduced to explain this observation ranging from ethnic genetic differences to changes in environmental exposures due to migration.^7-9^ Recent hypotheses point to differences in vitamin D metabolism through its receptor, VDR, which is a known transcription factor that plays a role in cell cycle regulation and apoptosis, synthesis, or its utilization in the body.^10,11^ Several studies have reported an inverse correlation between VDR expression and PCa risk and progression.^12, 13^

Pathological staging and microscopic grading of PCa are widely accepted as major morphology-driven prognostic factors of the disease. The tumor grade, which is denoted with a Gleason score, is the strongest clinical predictor of disease progression^14^ and correlates significantly with the local extent of the disease, lymph node, and bone metastasis, response to various therapies, and overall disease outcome.^13^

However, the clinicopathological correlates of VDR expression in PCa are not fully understood. This study explored the relationship between VDR expression, patient and tumor characteristics, and patterns of VDR expression dysregulation.

## Materials and method

### Prostate cancer cohort

This study retrospectively analyzed the clinical and genomic data of 497 PCa cases from The Cancer Genome Atlas (TCGA) database.^14^ Genomic and clinicopathological data were retrieved from the National Cancer Institute's Genomic Data Commons repository. TCGA cohort comprised 497 primary PCa cases with clinicopathological (including androgen deprivation therapy [ADT] outcome and overall and progression-free survival indices), RNASeq, chromosomal copy number segment, methylation, and somatic mutation data. Between 393 and 497 cases had data for clinicopathological indices; mRNA expression (by RNASeq) and chromosomal copy number segment (Affymetrix SNP 6.0 genotyping array) data were available for 497 cases while between 322 and 497 cases had methylation data (by methylation array on the Illumina Human Methylation 450 platform) for individual methylation loci.

### Data retrieval and data processing

Data retrieval was accomplished using codes and scripts that were written in Linux command. The PCa cohort had data for clinicopathological indices including age, race/ethnicity, disease stage, ADT response, overall and disease-free survival, and genomic data (e.g., methylation beta values); however, data on sunlight exposure were unavailable. Tumors were classified as localized disease (stages T1–T2c) or advanced disease (stages T3a–4) based on pathological stages. Prognostic grading was dichotomized into “good prognosis” (grade groups 1–2), “intermediate prognosis,” and “poor prognosis” (grade groups 4–5). Patients were categorized into two age groups: younger (<60 years) and older (≥60 years). PCa gene expression data sets for gene set enrichment analyses (GSEAs) were prepared using Linux-based scripts. The phenotype files were prepared in an Excel spreadsheet and converted to cls.

### Gene set enrichment analysis

GSEA was used to assess the biological relevancies of VDR expression in the PCa cohort. GSEA was performed to determine whether VDR-high and VDR-low cases had differential enrichment for relevant biological pathways. GSEA was accomplished with the Molecular Signature Database (MSigDB) hallmark (PI3K-AKT-MTOR and G2M checkpoint, TGFB, EMT, E2F, androgen response, glycolysis pathway, IFN-γ, IFN-α, ER, and apoptosis) and KEGG pathway (cell cycle and MAPK signaling, TNFR1, DEATH pathway, and MET) gene sets.^15,16^ GSEA ranks genes within the set and determines which members of the gene sets are at the top of the rank and how these gene sets show correlation with the phenotype. The maximum deviation from zero observed during the random permutations (permutational testing) is calculated and is the gene enrichment score (ES).^15,17^ This ES is higher when a gene in the set is observed and low when not available and is thus used to check the extent of association between VDR-low and VDR-high expression and PCa features. The normalized enrichment score is determined for each gene set.^15^ A gene set is considered to be statistically related to the phenotype if the nominal *P*-value is <0.05 and false discovery rate (FDR) is <0.25, as per MSigDB recommendations.

### Study approach

The clinicopathological significance of VDR in PCa was assessed using association and correlation analyses between VDR expression and clinicopathological indices. The relationship between VDR expression and genomic indices such as fraction genome altered (FGA) and tumor mutation burden (TMB) was also investigated using correlation analyses. Then, the biological significance of VDR expression was interrogated with GSEA. Furthermore, the dysregulation of VDR in PCa by copy number alteration and aberrant methylation was assessed using correlation and regression analyses.

### Statistical analyses

Clinicopathological and genomic and molecular data of interest were input to SPSS version 28 as categorical variables (age group, ethnic group, disease stage, pathological staging, Gleason grade, dichotomized VDR expression, DNA methylation, etc.) and continuous variables (age, Gleason score, etc.). Associations between two or more categorical variables were evaluated using the χ^2^ (or Fisher) test. Kaplan–Meier analysis was used to determine the relationship between VDR expression and overall and disease-free survival. Multiple linear regression analysis was used to determine which VDR methylation loci predicted VDR expression. All statistical analyses were performed in SPSS, and a *P*-value of <0.05 was regarded as significant. Benjamini–Hochberg correction was used to correct for multiple testing at an FDR of 0.05.

## Results

Of the 500 cases that were retrieved from TCGA cohort, 497 cases had VDR expression data. The median VDR expression level was used as a threshold to divide the cohort into VDR-high and VDR-low subsets. Following dichotomization, a total of 249 cases representing 50.1% had low VDR expression while 248 prostate cases (49.9%) had high VDR expression.

### High VDR expression is associated with adverse clinicopathological features of PCa

High VDR expression showed correlation with advanced pathological T and N stages, overall stage (TNM), high Gleason score, Gleason primary and secondary patterns, and Gleason grade group (*P* < 0.05), but not with patients' age or race (*P* > 0.05), as provided in Table 1. There was a positive correlation between VDR expression and Gleason score (r = 0.186, n = 497, *P* = 0.001). The most common primary Gleason pattern was 4 (n = 250), followed by pattern 3 (n = 197), pattern 5 (n = 49), and pattern 2 (n = 1). The chi-square test showed a statistically significant association between high VDR gene expressions and primary patterns 4 and 5, and low VDR expression is associated with pattern 3 [x^2^ (1) = 6.928; *P* = 0.010]. A similar trend was seen in the secondary patterns: Gleason pattern 4 was the most common (n = 235), followed by pattern 3 (n = 152) and pattern 5 (n = 110). High VDR expression also showed positive association with high secondary Gleason patterns [x^2^ (1) = 4.981; *P* = 0.026]. Similarly, high VDR expression was associated with poor prognostic grade groups, positive lymph nodes, and presence of advanced disease. Furthermore, an association between VDR expression and therapy outcome was observed. VDR-high cases were more likely to have less than complete response or progressive disease to androgen deprivation therapy than VDR-low cases (Table 1). However, VDR expression showed no association with time to biochemical recurrence of tumor. Overall, the results demonstrated that high VDR expression was associated with the adverse clinicopathological features of PCa; hence, VDR may have oncogenic effects in the context of PCa.

**Table 1:** Relationship of some clinicopathological variables and VDR gene expression.

S/N	Variable	Low VDR expression (n)	High VDR expression (n)	*P*	FDR
1	Age			0.623	0.779
	≤60 y	109	140		
	>60 y	114	134		
2	Race			0.772	0.858
	African American/Africans	27	30		
	Caucasians	210	215		
3	Tumor stage			**0.012**	**0.02**
	Localized disease	76	59		
	Advanced disease	111	147		
4	Lymph node status			**0.010**	**0.02**
	Negative	162	158		
	Positive	26	50		
5	Metastases			1.000	1.000
	No distant metastases	226	229		
	Distant metastases	1	2		
6	ISUP grade group			**˂0.001**	**0.0045**
	GG1	28	16		
	GG2	82	64		
	GG3	52	49		
	GG4	30	35		
	GG5	57	84		
7	ISUP prognostic category			**0.002**	**0.007**
	≤GG2	110	80		
	GG3	52	49		
	≥GG4	87	119		
8	ADT response type 1			**0.008**	**0.02**
	Complete response	125	98		
	Other responses	15	29		
9	ADT response type 2			**<0.001**	**0.0045**
	Any response	129	98		
	Progressive disease	11	29		
10	Copy number alterations			0.462	0.66
	VDR deletion	4	4		
	VDR wildtype	244	243		
	VDR gain/amplification	1	1		

Statistically significant values are highlighted in bold.

ADT, androgen deprivation therapy; FDR, false discovery rate; GG, grade group; ISUP, International Society of Urological Pathology.

### VDR expression versus genomic features of PCa

Although a positive correlation between FGA and VDR expression (r = 0.100, n = 491, *P* = 0.027) was found using correlation analyses, the independent-sample *t* test did not show any statistically significant association between the qualitative (high vs low) VDR expression and FGA [t (489) = −1.381, *P* = 0.168]. Nonsynonymous TMB did not show any statistically significant correlation with VDR expression (r = −0.001, n = 463, *P* = 0.988).

### VDR expression exhibited differential enrichment of biological pathways

**Figure 1.**
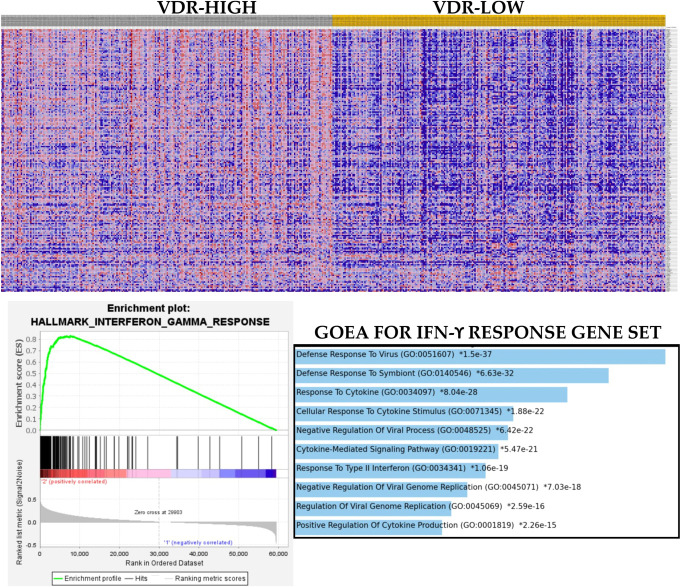
Heat map, enrichment plot, and ontology enrichment bar graph showing the enrichment of the hallmark interferon-gamma response gene set in the VDR-high subset of PCa. Gene Ontology enrichment analysis with bar graph production was accomplished with online Enrichr environment (https://appyters.maayanlab.cloud/Enrichment_Analysis_Visualizer/).

**Figure 2.**
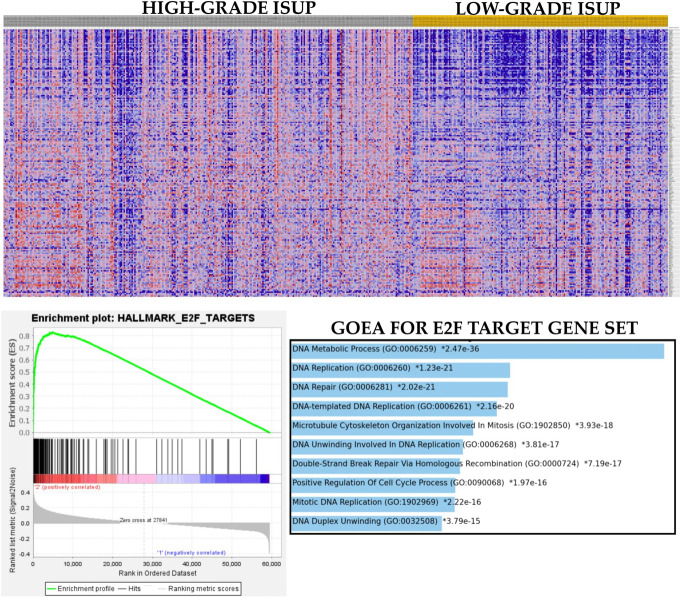
Heat map, enrichment plot, and ontology enrichment bar graph showing the enrichment of the hallmark E2F target gene set in the high-grade Gleason prognostic group subset of PCa. Gene Ontology enrichment analysis with bar graph production was accomplished with online Enrichr environment (https://appyters.maayanlab.cloud/Enrichment_Analysis_Visualizer/).

To validate the proposition that VDR may have oncogenic, rather than tumor suppressor, roles in PCa, GSEA was performed with hallmark tumor biology gene sets. The results showed that there was differential enrichment of biological pathways between the two VDR subsets of PCa. For example, while the VDR-high subset exhibited enrichment of oncogenic pathways such as the MAPK signaling, the epithelial–mesenchymal transition, the TGFB and TNFR1 signaling, cell proliferation and cell cycle pathways, and the glycolysis pathway (Table 2, Fig. 1), the VDR-low subset showed enrichment of oxidative phosphorylation, androgen response, and fatty acid metabolism pathways (Table 2). The result demonstrated that, in comparison with the VDR-low subset, the VDR-high cases were associated with more aggressive tumor biology, thereby lending credence to the proposition that VDR may function as an oncogene in PCa. Next, to assess whether these pathways may be related to the adverse clinicopathological features of PCa, we performed a Gleason grade–based GSEA. The results showed that the cases with high Gleason grades were enriched for 13 of the 20 pathways identified for the VDR-high subset (Table 3, Fig. 2), thereby supporting the notion that VDR signaling was associated with adverse clinicopathological indices of PCa.

**Table 2:** Gene set enrichment analysis for VDR-high and VDR-low subsets of prostate cancer.

	Size	ES	NES	NOM *P*	FDR q-val	FWER *P*	Rank at max
HALLMARK_INTERFERON_GAMMA_RESPONSE.v2023.2.Hs.grp	199	0.83	2.62	0	0	0	7645
HALLMARK_INTERFERON_ALPHA_RESPONSE.v2023.2.Hs.grp	97	0.83	2.52	0	0	0	7673
HALLMARK_INFLAMMATORY_RESPONSE.v2023.2.Hs.grp	200	0.78	2.47	0	0	0	5842
HALLMARK_EPITHELIAL_MESENCHYMAL_TRANSITION.v2023.2.Hs.grp	200	0.68	2.15	0	0	0	11971
HALLMARK_G2M_CHECKPOINT.v2023.2.Hs.grp	200	0.67	2.13	0	0	0	12641
HALLMARK_APOPTOSIS.v2023.2.Hs.grp	161	0.67	2.12	0	0	0	11609
HALLMARK_E2F_TARGETS.v2023.2.Hs.grp	200	0.65	2.06	0	0	0	12299
HALLMARK_TGF_BETA_SIGNALING.v2023.2.Hs.grp	54	0.73	2.05	0	0	0	5962
BIOCARTA_DEATH_PATHWAY.v2023.2.Hs.grp	29	0.78	2.03	0	0	0	6276
BIOCARTA_TNFR1_PATHWAY.v2023.2.Hs.grp	29	0.77	2.03	0	0	0	6945
BIOCARTA_MAPK_PATHWAY.v2023.2.Hs.grp	81	0.66	1.96	0	0	0	7176
KEGG_APOPTOSIS_NETWORK.Hs.grp	40	0.69	1.92	0	0	0	5946
BIOCARTA_MET_PATHWAY.v2023.2.Hs.grp	33	0.72	1.91	0	0	0	8308
HALLMARK_ESTROGEN_RESPONSE_EARLY.v2023.2.Hs.grp	198	0.59	1.87	0	0	0.001	7187
HALLMARK_ESTROGEN_RESPONSE_LATE.v2023.2.Hs.grp	198	0.55	1.73	0	0	0.002	10489
HALLMARK_PROTEIN_SECRETION.v2023.2.Hs.grp	96	0.54	1.66	0.001	0.001	0.012	11123
BIOCARTA_CELLCYCLE_PATHWAY.v2023.2.Hs.grp	23	0.66	1.65	0.001	0.001	0.015	11968
KEGG_CELL_CYCLE_PATHWAY.Hs.grp	23	0.66	1.65	0.003	0.001	0.015	11968
HALLMARK_GLYCOLYSIS.v2023.2.Hs.grp	200	0.5	1.59	0	0.002	0.041	7281
HALLMARK_BILE_ACID_METABOLISM.v2023.2.Hs.grp	112	0.34	1.07	0.36	0.344	1	13275
HALLMARK_OXIDATIVE_PHOSPHORYLATION.v2023.2.Hs.grp	200	−0.54	−2.54	0	0	0	5324
HALLMARK_ANDROGEN_RESPONSE.v2023.2.Hs.grp	100	−0.37	−1.6	0	0.014	0.015	6055
HALLMARK_FATTY_ACID_METABOLISM.v2023.2.Hs.grp	157	−0.32	−1.45	0	0.023	0.037	5539

ES, enrichment score; FDR, false discovery rate; FWER, familywise error rate; Max, maximum; NES, normalized enrichment score; NOM, nominal; *P*, *P*-value; q-val, q-value.

**Table 3:** Gene set enrichment analysis of high-grade versus low-grade prostate cancer.

	Size	ES	NES	NOM *P*	FDR Q-val	FWER *P*	Rank at max
HALLMARK_E2F_TARGETS	200	0.83	3.32	<0.001	<0.001	<0.001	4841
HALLMARK_G2M_CHECKPOINT	200	0.77	3.07	<0.001	<0.001	<0.001	6941
BIOCARTA_CELLCYCLE_PATHWAY	23	0.77	2.17	<0.001	<0.001	0.001	6717
BIOCARTA_MAPK_PATHWAY	81	0.56	2.03	<0.001	<0.001	0.027	9969
HALLMARK_INTERFERON_ALPHA_RESPONSE	97	0.55	2.02	<0.001	<0.001	0.029	16306
HALLMARK_INTERFERON_GAMMA_RESPONSE	198	0.49	1.96	<0.001	0.001	0.084	18568
HALLMARK_GLYCOLYSIS	200	0.46	1.84	<0.001	0.005	0.428	10528
BIOCARTA_TNFR1_PATHWAY	29	0.56	1.68	0.01	0.024	0.982	9741
HALLMARK_TGF_BETA_SIGNALING	54	0.49	1.68	0.006	0.024	0.983	11968
BIOCARTA_DEATH_PATHWAY	29	0.52	1.58	0.014	0.052	1	11855
HALLMARK_EPITHELIAL_MESENCHYMAL_TRANSITION	200	0.39	1.56	<0.001	0.058	1	7450
HALLMARK_ESTROGEN_RESPONSE_LATE	198	0.33	1.32	0.028	0.199	1	8658
HALLMARK_INFLAMMATORY_RESPONSE	200	0.33	1.3	0.041	0.209	1	11614
HALLMARK_APOPTOSIS	161	0.33	1.28	0.062	0.227	1	11154
HALLMARK_BILE_ACID_METABOLISM	112	0.33	1.26	0.094	0.24	1	10952
HALLMARK_PROTEIN_SECRETION	96	0.34	1.25	0.112	0.253	1	9811
BIOCARTA_MET_PATHWAY	33	0.34	1.07	0.354	0.461	1	9806

ES, enrichment score; FDR, false discovery rate; FWER, familywise error rate; Max, maximum; NES, normalized enrichment score; NOM, nominal; *P*, *P*-value; q-val, q-value.

### VDR expression and survival profile

Overall survival data were available for 491 of 497 cases. The follow-up duration for the cohort was 120 months. Within this period, 91 of 491 patients had died, comprising 45 VDR-low cases and 46 VDR-high cases. The remaining 400 of 491 were alive at the end of the study period, comprising 200 each of the VDR subsets. The mean survival time for the VDR-low subset was 85.528 (76.508–95.547) months; for the VDR-high subset, it was 83.798 (74.108–93.488) months. The log-rank test showed no significant difference in the overall survival between VDR-low and VDR-high cases (X^2^ = 0.000; *P* = 0.988). Disease-free survival showed exactly the same profile as overall survival. No data were available for progression-free survival for this cohort.

### VDR expression is deregulated by aberrant methylation at VDR promoter regions in PCa

**Figure 3.**
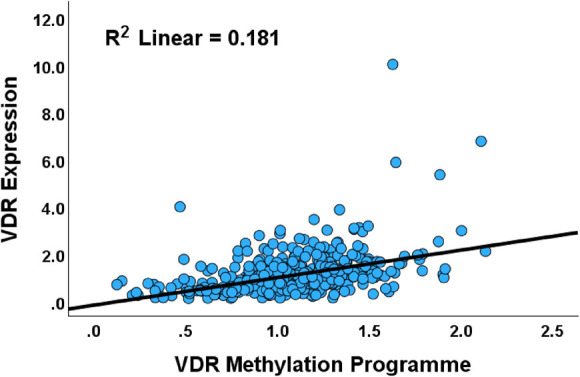
Scatterplot showing the overall relationship between VDR expression and the cumulative VDR methylation program in the PCa cohort. The VDR methylation program includes methylation loci that display canonical methylation-expression correlation and those that have paradoxical relationship with VDR expression.

The copy number status of VDR in the PCa cohort was mainly wildtype (n = 487), followed by gene deletions (n = 8) and gene gain/amplification (n = 2). The copy number alteration did not show any association with VDR expression in PCa (X^2^ = 0.00, *P* = 1.00). Methylation scores (beta values) were available for all 497 cases with VDR expression data. Multiple linear regression was used to evaluate whether VDR methylation predicts VDR expression. The analysis revealed a correlation between methylation status and VDR expression for the loci VDR_cg06369854, VDR_cg10037049, VDR_cg10592901, VDR_cg16321474, and VDR_cg22833603 (Table 4, Fig. 3). Notably, while VDR_cg10037049, VDR_cg16321474, and VDR_cg22833603 exhibited an inverse relationship with VDR expression, the remaining two loci (VDR_cg10592901, VDR_cg06369854) showed a direct or positive correlation with VDR expression. These findings suggest that VDR expression is mainly deregulated by epigenetic mechanisms.

**Table 4:** Multiple linear regression model of VDR methylation loci in prostate cancer.

R	R^2^	Adjusted R^2^	SE of estimate
0.431	0.186	0.177	0.736

Coefficients				
	Unstandardized coefficients		t	*P*
	B	SE		
(Constant)	6.386	0.912	7.005	<0.001
VDR_cg06369854	2.094	0.668	3.134	0.002
VDR_cg10037049	−1.953	0.288	−6.776	<0.001
VDR_cg10592901	1.353	0.345	3.919	<0.001
VDR_cg16321474	−1.769	0.527	−3.356	0.001
VDR_cg22833603	−4.880	1.069	−4.564	<0.001

ANOVA			
	df	F	*P*
Regression	5	20.578	<0.001
Residual	450		
Total	455		

SE, standard error.

## Discussion

The findings of this study clearly showed that high VDR expression was associated with some adverse clinicopathological indices such as high Gleason scores, poor prognostic category, advanced disease, and poor therapy response. It is also interesting to note that the main copy number variant of VDR is the wildtype (or neutral CNV), and that the major mechanism of VDR dysregulation is through epigenetics. Our study not only found that VDR expression was significantly associated with adverse clinical indices but also further provided evidence, through GSEA, of the basis of such association. Clearly, VDR expression, based on this study's findings, behaved as an oncogene not only through its clinicopathological correlations but also through its correlation with established biological pathways of tumorigenesis and tumor progression.

Studies have demonstrated that the activity of CYP 27B1 enzyme responsible for bioactivation of calcitriol is repressed in PCa cells in culture when compared with nonmalignant prostate epithelium.^18^ VDR is a ligand-inducible transcription factor and a member of the nuclear receptor super family. Calcitriol is believed to induce cell cycle arrest after binding to VDR by blocking the G2-M phase and G1-S arrest.^18^ Thus, absence of vitamin D will promote proliferation of PCa cells. Ligand-dependent transcriptional activators have been shown to permit conditional gene expression in response to small-molecule stimuli.^19^

There is an inverse correlation between VDR expression and malignancy as has been reported in some studies.^20,21^ While our study indicated that high VDR expression is positively correlated with adverse PCa features, Hendrickson et al demonstrated that high VDR expression had significant association with low PSA at diagnosis, low Gleason score, and less advanced tumor stage.^21^ It is interesting to note that vitamin D has shown antiproliferative activity in PCa. The effect of active vitamin D is mediated by VDR, which has been demonstrated to negatively regulate androgenic signaling, and that high VDR signaling reduces PCa progression.^22^ Likewise, some alleles of VDR have shown PCa risk reduction, for example, *FokI FF*, *Ff*, and *TaqI tt*.^23,24^ Nezbedova et al in a review showed that calcitriol is important in transactivation of the VDR genes with subsequent suppression of proliferation.^20^ On the contrary, the relationship between VDR signaling and the clinical features of PCa could be complex. For example, some polymorphic variants of VDR such as *BsmI and ApaI* are associated with poor prognostic factors and are predictive of lethal cancer.^25^ The report by Beckett et al showed that in the presence of *BsmI,* high UV exposure would lead to increased methylation density of the VDR, leading to dysregulation of the antiproliferative activity of the *vitamin D/*VDR system.^26,27^ Doig et al^10^ also showed that there can be selective attenuation of response of VDR to the antiproliferative effect of binding to vitamin D through transcriptional reprogramming. Under such circumstances, VDR expression would not suppress proliferation but could in fact be associated with promotion of proliferation and lethal clinicopathological variables in PCa, similar to this study's findings. These complexities in the *vitamin D/*VDR system might explain why despite the positive correlation between high VDR expression and unfavorable pathological parameters, it did not affect survival in this cohort.

The discordance of our findings with those of other studies is probably one of the gray areas of the pathogenesis of PCa that could hinder the effective utility of *vitamin D/*VDR system for the management of PCa. Cancer cells have shown ability to epigenetically alter gene expressions that would prevent the malignant cells from thriving, thereby allowing for unchecked carcinogenesis.^28^ Hence, despite the high VDR expression, the prostate cells will be insensitive to the antiproliferative control of vitamin D or might rewire the cellular signaling network to use VDR for tumor promotion.^28^ Likewise, polymorphisms in VDR as against general protein expression is associated with PCa risk.^23,29^

These VDR polymorphisms show varying distributions in different populations; this differential distribution has been postulated to result from varying sun exposure levels of the different populations.^30^ It was also demonstrated that the circulating levels of serum vitamin D showed an inverse correlation with VDR expression.^12^ We do not have the serum vitamin D levels in this cohort to draw correlation, of probably low plasma vitamin D inducing higher expression of VDR.

Inadequate sun exposure with consequent low calcitriol levels has been implicated with higher risks of PCa.^31^ The African skin in temperate regions is at a disadvantage as it would require higher volume of sunlight to produce calcitriol.^28^ Sunlight exposure has an inverse relationship with PCa mortality, as even PCa risk is greater in men with lower levels of vitamin D.^32^ VDR expression did not show any significant variations based on the race/ethnic origin of patients with PCa in this cohort, perhaps because of disparity in representation of some ethnicities in TCGA.

PCa is most common in older age groups and some studies have evaluated the age-dependent variation in PCa, but findings have been inconsistent.^12,33,34^ Cells with higher VDR content would naturally be more responsive to the antiproliferative activity of vitamin D; thus, we would expect higher levels in the younger age groups. That is not the case, however, in this cohort as there was no difference in VDR expression among the age groups of the patients. This is probably because these are all patients with PCa and would expectedly have low serum vitamin D activity inasmuch as a review by Merchan et al^28^ postulates that in late-stage cancers, the vitamin D/VDR system is probably less functional.

This study found that VDR expression in PCa is dysregulated by methylation mechanisms. A mixed methylation-expression pattern was found in this study, wherein some methylation loci exhibited the canonical inverse correlation with gene expression, whereas some others showed paradoxical methylation patterns.^26,27,35^ Previous studies have shown that the vitamin D and VDR system is corrupted by hypermethylation mechanisms of the genes.^28^ Other reported mechanisms of VDR dysregulation and suppression involve histone deacetylation.^27^

The study was limited by the available clinicopathological characteristics on this cohort. For example, data on an important prognostic variable, such as positive resection margins, were not available for correlation with VDR. Likewise, most of the patients were Caucasians and living in temperate regions, which limited our ability to correlate race and environmental influences with VDR expression. Despite these several limitations, the study raises insights (genomic analyses) into the impact of vitamin D receptor expression in prostate cancer that may interest readers.

In conclusion, VDR expression was higher in patients with advanced disease, elevated Gleason scores, poor prognostic grades, and poor drug response profile. However, it showed no association with overall or disease-free survival in this cohort. No significant variations in VDR expression were observed based on the age or race/ethnic origin of patients with prostate cancer. While high VDR expression was associated with unfavorable prognostic pathological and biological parameters, it did not affect disease-free survival.

### Future prospects

The biological activity of VDR in prostate carcinogenesis and the potential of targeting the pathway for therapy could be explored by future studies.
